# A Facile Method to Construct MXene/CuO Nanocomposite with Enhanced Catalytic Activity of CuO on Thermal Decomposition of Ammonium Perchlorate

**DOI:** 10.3390/ma11122457

**Published:** 2018-12-04

**Authors:** Haifeng Zhao, Jing Lv, Junshan Sang, Li Zhu, Peng Zheng, Greg. L. Andrew, Linghua Tan

**Affiliations:** 1 National Special Superfine Power Engineering Research Center, Nanjing University of Science and Technology, Nanjing 210094, China; zhf950613@163.com (H.Z.); lvjing9487@163.com (J.L.); 15151853730@163.com (L.Z.); zhengpeng97@163.com (P.Z.); 2 Gansu Yinguang Chemical Industry Group Co., Ltd., Baiyin 730900, China; sangjunshan123@163.com; 3 College of Medical, Veterinary, and Life Sciences, University of Glasgow, G12 8QQ Glasgow, UK; greg.l.andrew@gmail.com

**Keywords:** carbides, MXene, layered compounds, transition metal oxide, composite, thermal decomposition, ammonium perchlorate

## Abstract

In this work, a mixing-calcination method was developed to facilely construct MXene/CuO nanocomposite. CuO and MXene were first dispersed in ethanol with sufficient mixing. After solvent evaporation, the dried mixture was calcinated under argon to produce a MXene/CuO nanocomposite. As characterized by X-ray diffraction (XRD), field-emission scanning electron microscopy (FESEM), and X-ray photoelectron spectra (XPS), CuO nanoparticles (60–100 nm) were uniformly distributed on the surface and edge of MXene nanosheets. Furthermore, as evaluated by differential scanning calorimetry (DSC) and thermal gravimetric analysis (TGA), the high-temperature decomposition (HTD) temperature decrease of ammonium perchlorate (AP) upon addition of 1 wt% CuO (hybridized with 1 wt% MXene) was comparable with that of 2 wt% CuO alone, suggesting an enhanced catalytic activity of CuO on thermal decomposition of AP upon hybridization with MXene nanosheets. This strategy could be further applied to construct other MXene/transition metal oxide (MXene/TMO) composites with improved performance for various applications.

## 1. Introduction

Since the first report of Ti_3_C_2_ in 2011, MXenes have gained significant attention as a new family of 2D transition metal carbides or nitrides. The production of MXenes (M_n+1_X_n_T_x_, M for transition metal element, X for carbon or nitrogen, T for -OH, -O and -F) can be achieved through selective etching of A (group IIIA or IVA elements) layers from ternary metal carbides or nitrides (MAX phase) [1,2,3]. Considerable properties have since been reported, such as graphene-like layered structure, electrical conductivity, hydrophilicity, and flexibility [4]. MXenes have been widely studied for their applications in many fields; for instance, MXenes have been investigated as electrode materials in Li-ion batteries [5,6] as well as supercapacitors [7,8,9], adsorption materials [10,11], hydrogen storage materials [12], and catalysts [13,14,15].

Various methods have been adopted to hybridize transition metal oxide (TMO) with MXene to prepare MXene/TMO composites, inherent of each material’s unique properties, for multifaceted applications. However, most commonly adopted methods, such as hydrothermal [16,17] and precipitation methods [10,18], need precise control of reaction conditions and sometimes a long reaction time and a large amount of solvent are necessary. Such methods are unfavorable for the efficient production and environment protection. Liu et al. since reported the self-assembly of TiO_2_ nanorods and SnO_2_ nanowires with MXene nanosheets under ambient conditions by utilizing their well-developed “surface energy compensation strategy” [14]. Upon transferring the MXene well-dispersed solution to its poor solvent, in which TMO nanoparticles were well dispersed, TMO nanoparticles tend to deposit on the naked surface of MXene nanosheets to minimize surface energy stabilized through van der Walls interactions [19,20]. It should be noted that the premodification of an organic layer on TMO nanostructures is often necessary for this method, for the purpose of improving the organic dispersibility of TMO.

In this work, a mixing-calcination method to simply construct a MXene/CuO nanocomposite was developed. The catalytic effect of the MXene/CuO nanocomposite on the thermal decomposition of ammonium perchlorate (AP), the most common oxidant in composite solid propellants, was examined to demonstrate the enhanced catalytic activity of CuO upon hybridization with MXene nanosheets.

## 2. Materials and Methods

All the reagents were purchased from commercial sources and were utilized as received without further purification. The crystalline phases, morphology, and surface chemical composition of the samples were studied by employing X-ray diffraction (XRD) (D8 ADVANCE, Bruker AXS GmbH, Karlsruhe, Germany, Cu Kα irradiation, λ = 0.15406 nm, 2θ = 5~80°), field-emission scanning electron microscopy (FESEM) (Zeiss MERLIN Compact, Jena, Germany), and X-ray photoelectron spectra (XPS) (ESCALAB250Xi, Thermo Fisher Scientific., Rockford, Tempe, AZ, USA, C1s line 284.8 eV as a reference for calibration), respectively.

Ti_3_C_2_T_x_ MXene preparation. Ti_3_AlC_2_ (2 g) was slowly added to 40 mL of 40% HF solutions and the reaction mixture was stirred at 60 °C for 18 h. The solids in the solution were collected by centrifuge, washed with deionized water, and lyophilized. During the preparation, the following reaction equation was followed: Ti_3_AlC_2_ + 3HF → AlF_3_ + 3/2H_2_ + Ti_3_C_2_ [2].

MXene/CuO composite preparation. MXene/CuO nanocomposites containing different amounts of CuO (5, 10, 30, and 50 wt%) were prepared and labeled as MXene/x% CuO (x = 5, 10, 30, and 50). Typical preparation of MXene/10% CuO catalyst was as follows: 0.2 g of Ti_3_C_2_T_x_ MXene power was completely dispersed in ethanol by sonication for 10 min, followed by the addition of 0.022 g of CuO into the above solution. After a further 10 min of sonication and ethanol evaporation at 60 °C for 1 h, MXene/CuO was collected and calcined in a tube furnace under argon at 300 °C for 1 h.

AP thermal decomposition. The experiments were conducted according to our previously published procedure [21].

## 3. Results and Discussions

**Figure 1:**
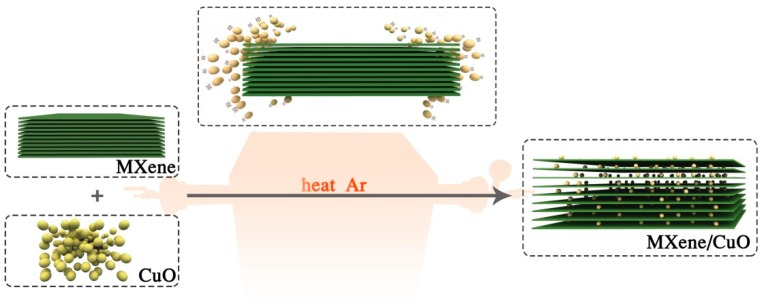
Illustration of mixing-calcination strategy for the facile construction of a MXene/CuO nanocomposite.

The developed mixing-calcination method for the facile construction of MXene/CuO nanocomposite is presented in Figure 1. Ti_3_C_2_T_x_ (MXene) was first prepared by using HF as an etchant to remove Al layers from Ti_3_AlC_2_. After achieving suitable dispersion with long-term stability in ethanol with the help of sonication [22], CuO was added to the above solution with sufficient sonication. Following solvent evaporation of ethanol, the obtained mixture was calcinated under argon to produce MXene/CuO nanocomposite.

**Figure 2:**
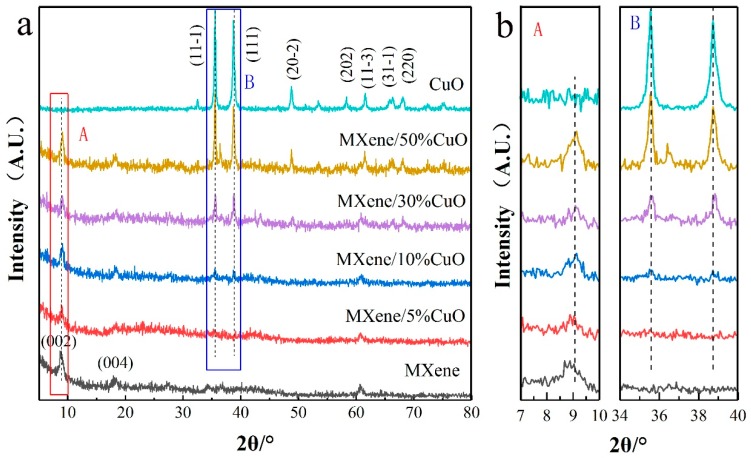
(**a**) X-ray diffraction (XRD) diffraction pattern of MXene, CuO, and MXene/x% CuO (x = 5, 10, 30, and 50); (**b**) Enlarged profiles of the selected area in Figure 2a.

The crystal structures of MXene, CuO, and MXene/CuO nanocomposites with various CuO contents were studied by using XRD. As shown in Figure 2, the diffraction peaks with 2θ values at 9.0° and 18.32° could be attributed to (002) and (004) planes of MXene, suggesting successful synthesis of MXene [2,23]. The peaks at 35.54°, 38.71°, 48.72°, 58.26°, 61.52°, 66.22°, and 68.12° in the CuO pattern could be indexed to (11-1), (111), (20-2), (202), (11-3), (31-1), and (220) planes of monoclinic CuO (JCPDS 48-1548) [24], respectively. Diffraction peaks corresponding to both MXene and CuO were observed in the pattern of MXene/CuO nanocomposite, and the peak intensity increased with the increased content of CuO, demonstrating the successful fabrication of the MXene/CuO nanocomposite.

**Figure 3:**
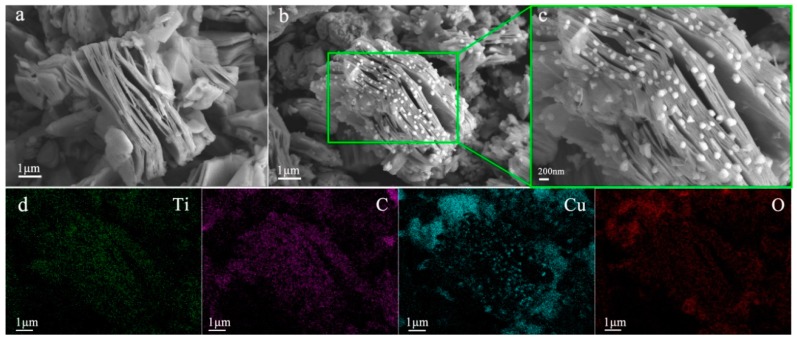
Field-emission scanning electron microscopy (FESEM) images of MXene (**a**), MXene/50% CuO nanocomposite (**b**,**c**), and elemental mapping results (**d**).

FESEM was employed to observe the morphology and structure of MXene/CuO nanocomposite by taking MXene/50% CuO as a representative. As shown in Figure 3a, a typical exfoliated morphology of separated Ti_3_C_2_T_x_ layers was observed, indicating the successful exfoliation of Ti_3_AlC_2_ [25]. The FESEM images of MXene/50% CuO exhibited CuO nanoparticles with 60–100 nm diameters, were randomly deposited on the surface and edge of the MXene nanosheets (Figure 3b,c), and were stabilized through van der Walls interactions [20]. Elemental distributions of C, Ti, Cu, and O in the MXene/CuO nanocomposite were determined by means of energy dispersive spectroscopy (EDS) area scanning (Figure 3d). The observed maps of C, Ti, Cu, and O demonstrated successful hybridization of CuO nanoparticles on MXene nanosheets. Furthermore, the similar profile of Cu and O also suggested a homogeneous distribution of CuO nanoparticles on MXene nanosheets.

**Figure 4:**
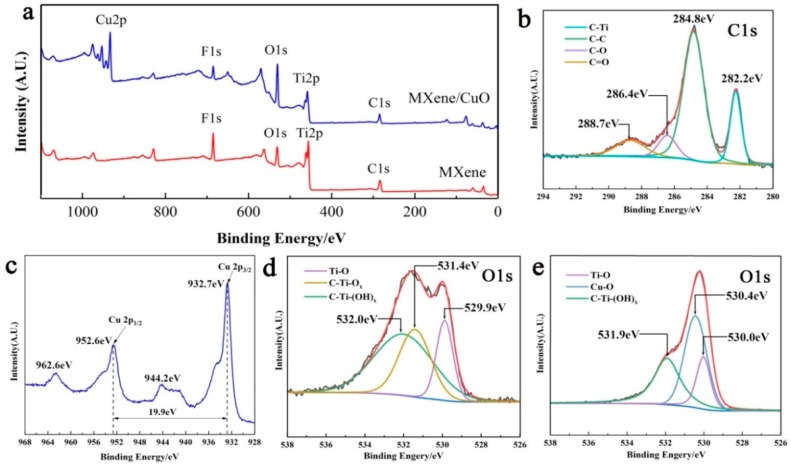
X-ray photoelectron spectra (XPS) of the MXene and MXene/50% CuO nanocomposite: full scan (**a**), C 1s spectra of MXene/50% CuO (**b**), Cu 2p spectra of MXene/50% CuO (**c**), O 1s spectra of MXene (**d**), and MXene/50% CuO (**e**).

Figure 4a demonstrates the XPS spectra of the MXene and MXene/50% CuO nanocomposite. In the spectrum of MXene, the peaks representative of C, Ti, O, and F could be clearly observed. As for the MXene/50% CuO nanocomposite, apart from the peaks corresponding to C, Ti, O, and F, peaks corresponding to Cu were also found in the spectrum, demonstrating the successful hybridization of CuO with MXene. The presence of C–Ti bonds at 282.2 eV in the high-resolution spectra of C 1s (Figure 4b) demonstrated the retained structure of MXene after hybridization with CuO [26]. The two main binding energy peaks at 932.7 and 952.6 eV with a peak splitting of 19.9 eV in the high-resolution spectrum of Cu 2p (Figure 4c) could be ascribed to Cu 2p_3/2_ and Cu 2p_1/2_ [27,28], respectively. Moreover, the satellite peaks at 944.2 and 962.6 eV further confirmed the existence of CuO [29]. The coexistence of the peaks corresponding to Ti–O, C–Ti–(OH)x, and Cu–O bonds in the high-resolution spectra of O 1s of MXene/50% CuO composites further demonstrated the successful construction of MXene/CuO through the developed mixing-calcination strategy (Figure 4d,e) [14,30].

**Figure 5:**
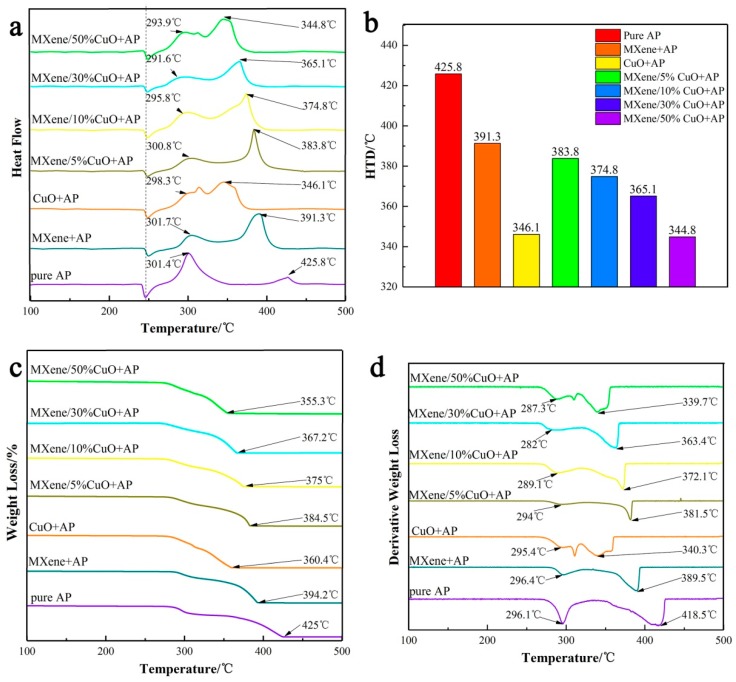
Differential scanning calorimetry (DSC) curves of ammonium perchlorate (AP) in the absence and presence of 2 wt% catalysts (**a**), histogram of the corresponding high-temperature decomposition (HTD) of AP from DSC results (**b**), thermal gravimetric analysis (TGA) and differential TGA (DTGA) curves of AP in the absence and presence of 2 wt% catalysts (**c**,**d**).

We further evaluated the catalytic effect of the MXene/CuO nanocomposite on thermal decomposition of AP by referring to our reported method [21]. Differential scanning calorimetry (DSC) and thermal gravimetric analysis (TGA) were employed to determine the decomposition behavior of AP in the absence and in the presence of 2 wt% CuO, 2 wt% MXene, and 2 wt% MXene/x% CuO (x = 5, 10, 30, and 50), respectively. One possibility considered was that the distribution of CuO nanoparticles could be improved on the surface of MXene, resulting in an increase of active sites of CuO during the catalytic process. Alternatively, with good thermal conductivity and lamella structure of MXene, the heat transfer and the gas phase absorption of NH_3_ and HClO_4_ could possibly be promoted. As shown in Figure 5a,b, upon addition of 2 wt% MXene and 2 wt% CuO, the high-temperature decomposition (HTD) temperatures of AP were decreased by 34.5 and 79.7 °C, respectively. The exhibited decrease suggests a good catalytic activity of the two materials alone. When treated with 2 wt% MXene/x% CuO (x = 5, 10, 30, and 50), the HTD temperature decrease of AP exhibited a CuO-content-dependent manner, and the highest HTD temperature decrease (81.0 °C for 1 wt% CuO hybridized with 1 wt% MXene) was observed when the content of CuO was 50%, which was comparable with that of 2 wt% CuO alone, indicating that the catalytic activity of CuO was enhanced upon its hybridization with MXene nanosheets. The TGA and differential TGA (DTGA) curves in Figure 5c,d show that upon addition of the 2 wt% MXene/50% CuO nanocomposite, the final weight-loss temperature of AP was reduced by 78.8 °C. In contrast, the addition of 2 wt% CuO and 2 wt% MXene alone reduced the final weight-loss temperature of AP by 78.2 and 29 °C, respectively. The observation is consistent with those from DSC analysis, further demonstrating an enhanced catalytic activity of CuO following hybridization with MXene nanosheets.

## 4. Conclusions

In conclusion, a MXene/CuO nanocomposite was constructed through a facile mixing-calcination method. CuO nanoparticles were uniformly loaded on the surface and edge of MXene nanosheets, evidenced by means of XRD, FESEM, and XPS. The catalytic activity of CuO on thermal decomposition of AP was enhanced upon hybridization with MXene nanosheets, as concluded from the comparable HTD temperature decrease of AP upon addition of 1 wt% CuO (hybridized with MXene) to that of 2 wt% CuO alone. Our strategy could be applied to fabricate further MXene/TMO composites with improved performance of TMO for various applications.
